# Association between circadian physical activity patterns and mortality in the UK Biobank

**DOI:** 10.1186/s12966-023-01508-z

**Published:** 2023-09-01

**Authors:** Michael J. Stein, Hansjörg Baurecht, Anja M. Sedlmeier, Julian Konzok, Patricia Bohmann, Emma Fontvieille, Laia Peruchet-Noray, Jack Bowden, Christine M. Friedenreich, Béatrice Fervers, Pietro Ferrari, Marc J. Gunter, Heinz Freisling, Michael F. Leitzmann, Vivian Viallon, Andrea Weber

**Affiliations:** 1 Department of Epidemiology and Preventive Medicine, University of Regensburg, Franz-Josef-Strauss Allee 11, 93057 Regensburg, Germany; 2 Nutrition and Metabolism Branch, International Agency for Research On Cancer (IARC/WHO), Lyon, France; 3 Department of Clinical Sciences, Faculty of Medicine, University of Barcelona, Barcelona, Spain; 4 University of Exeter Medical School, Exeter, UK; 5 Novo Nordisk Research Center Oxford, Oxford, UK; 6 Department of Cancer Epidemiology and Prevention Research, Cancer Care Alberta, Alberta Health Services, Calgary, AB Canada; 7 Departments of Oncology and Community Health Sciences, Cumming School of Medicine, University of Calgary, Calgary, AB Canada; 8 Département Prévention Cancer Environnement, Centre Léon Bérard, Lyon, France; 9 INSERM U1296 Radiation: Defense, Health, Environment, Lyon, France

**Keywords:** Physical activity patterns, All-cause mortality, UK Biobank, Raw accelerometry

## Abstract

**Background:**

The benefit of physical activity (PA) for increasing longevity is well-established, however, the impact of diurnal timing of PA on mortality remains poorly understood. We aimed to derive circadian PA patterns and investigate their associations with all-cause mortality.

**Methods:**

We used 24 h PA time series from 96,351 UK Biobank participants aged between 42 and 79 years at accelerometry in 2013–2015. Functional principal component analysis (fPCA) was applied to obtain circadian PA patterns. Using multivariable Cox proportional hazard models, we related the loading scores of these fPCs to estimate risk of mortality.

**Results:**

During 6.9 years of follow-up, 2,850 deaths occurred. Four distinct fPCs accounted for 96% of the variation of the accelerometry data. Using a loading score of zero (i.e., average overall PA during the day) as the reference, a fPC1 score of + 2 (high overall PA) was inversely associated with mortality (Hazard ratio, HR = 0.91; 95% CI: 0.84–0.99), whereas a score of -2 (low overall PA) was associated with higher mortality (1.69; 95% CI: 1.57–1.81; p for non-linearity < 0.001). Significant inverse linear associations with mortality were observed for engaging in midday PA instead of early and late PA (fPC3) (HR for a 1-unit increase 0.88; 95% CI: 0.83–0.93). In contrast, midday and nocturnal PA instead of early and evening PA (fPC4) were positively associated with mortality (HR for a 1-unit increase 1.16; 95% CI: 1.08–1.25).

**Conclusion:**

Our results suggest that it is less important during which daytime hours one is active but rather, to engage in some level of elevated PA for longevity.

**Supplementary Information:**

The online version contains supplementary material available at 10.1186/s12966-023-01508-z.

## Introduction

Physical inactivity is a global concern, with 28% of the world’s population not attaining physical activity (PA) recommendations [1]. Such data are disconcerting because of robust evidence on the association of insufficient PA with increased premature mortality [2, 3].

PA is a complex construct, the valid measurement of which is challenging [4]. Most evidence on the PA and mortality relation stems from studies using PA self-report methods. Such methods provide data on PA type, dose, and timing but they suffer from PA measurement error [2]. Accelerometers are increasingly preferred as PA measure [5] because they show high validity [6] and quantify temporal nuances in movement behaviors [7]. However, raw accelerometry data show considerable within and between subject heterogeneity and the data volume and complexity are challenging to analyze [8]. Thus, most previous studies have relied on summary accelerometry output instead of comprehensively examining movement and rest profiles throughout the day [9].

Functional principal component analysis (fPCA), an extension of common PCA, is well-suited to analyze temporal patterns and thus represents a promising technique to identify circadian PA patterns. Most previous studies using fPCA have been limited by relying on device-specific summary metrics (‘activity counts’) [10–12] or small sample sizes [10, 12, 13]. Few studies have overcome these limitations and those that did showed that fPCs denoting lower or evening PA were positively associated with mortality among older men [14] and that fPCs were associated with socio-demographic characteristics and self-rated health [9].

In this study we aimed to go beyond what has been conducted in previous investigations since it is the first to examine fPCA-based PA patterns in relation to all-cause mortality in a large cohort of men and women.

## Methods

### Study population and data collection

The UK Biobank (UKB) is a prospective cohort study of > 500,000 UK participants aged 40–69 years when recruited between 2006–2010. The study collected data on sociodemographic and lifestyle factors, and extensive phenotypic information. The assessment visit included a touchscreen questionnaire, interviews, physical and functional measurements, and the collection of biologic samples. The UKB obtained ethics approval from the North West Multi-Centre Research Ethics Committee. All participants provided written informed consent [15].

### Physical activity data

For a subset of 103,669 participants, device-based PA data were available, measured in 2013–2015 using the Axivity AX3 (Newcastle Upon Tyne, UK) wrist-worn triaxial accelerometer for seven days. No participants were pre-excluded from the accelerometer study based on health problems. Participants with valid email addresses were randomly invited to participate in the accelerometer study and were informed that the accelerometer was to be worn continuously on the dominant wrist immediately upon receipt. The device was configured to activate shortly after its arrival and was deactivated seven days later. Subsequently, participants were asked to return the device to the coordinating center. Data processing was conducted by the UKB expert working group and is detailed elsewhere [16]. In brief, the Euclidean norm minus one (ENMO) was derived from raw data. ENMOs represent a summary metric of bodily acceleration measured in milligravity units (m*g*) interpretable as PA volume (Online Resource 1). Participants with data from at least 72 h and data for each one-hour period of the 24-h cycle (scattered over multiple days) were included, as recommended by the UKB expert working group (*N* = 96,665). Further, we excluded participants with average daily ENMOs above the 99.9 percentile (*N* = 97) and/or missing covariates (*N* = 217), leading to 96,351 participants (= PA pattern sample). We used the average hourly acceleration over the participant-specific recorded days, resulting in a 96,351 × 24 matrix.

### Functional principal component analysis

We used fPCA to reduce the dimensionality of the data and to derive PA patterns while retaining information on between-person variation. fPCA calculates components of PA time series data on which each participant scores with a certain loading. These fPCs, or eigenfunctions, depict the strongest and most important modes of variability in the PA data [17]. The loading score, or eigenvalue, reflects the extent to which a participant’s activity data follows a certain pattern [14]. Each participant contributes to each identified pattern, either positively (positive loading) or negatively (negative loading). We used fPCA through conditional expectation (principal analysis by conditional estimation, PACE), developed for sparse longitudinal data with only few repeated observations per subject [18].

We modeled ENMOs using linear regression, adjusted for age, sex, body mass index, and study center to obtain PA residuals. These were subsequently standardized and subjected to the fPCA. We used a Gaussian kernel smoother and the default for estimating the bandwidth (5% of the observed time range for the mean function; 10% for the covariance function). We tested the robustness of our results in sensitivity analyses using generalized cross-validation for bandwidth selection in conjunction with alternative kernel smoothings. The Epanechnikov kernel is (compared to Gaussian) a compact kernel ($\left|x-{x}_{0}\right| \le 1$) that minimizes (among all kernel smoothers) the asymptotic mean integrated squared error. The number of relevant components was determined using the elbow method, an explained total variability threshold of > 95%, and visual inspection of the eigenfunctions [19].

We used the R package *fdapace* to apply the fPCA [20].

### Cohort follow-up and ascertainment of mortality cases

Participants’ vital status was determined through linkage with routine health care data and national death registries [21]. Follow-up began at the baseline accelerometry measurement (June 2013 to December 2015) and ended at the date of complete follow-up (September 2021 for England/Wales or October 2021 for Scotland) [22], lost-to-follow-up, or date of death. All-cause mortality was considered the endpoint.

### Covariates

We identified potential confounding covariables *a priori* based on evidence-derived directed acyclic graphs [23] (Online Resource 2). Covariate details are given in Online Resource 3. Briefly, the main model was stratified by sex and study center and was further adjusted for prevalent diabetes and cardiovascular disease (CVD) obtained from hospital inpatient records pre-accelerometry, as well as baseline (2006–2010) information on smoking status, alcohol consumption status, socio-economic status, education level, sedentary behavior, and diet.

### Statistical analysis

Statistical analysis was conducted with complete data after removing missing covariate data (Online Resource 4). Participants with prevalent malignant cancer other than non-melanoma skin cancer (pre-accelerometry; cancer registry data) (*N* = 10,288) were excluded to minimize reverse causation [24]. Ultimately, we included 84,877 participants in our assessment of all-cause mortality (= mortality sample).

Cox proportional hazards regression with age as the underlying time metric [25] was used to estimate hazard ratios (HR) and corresponding 95% confidence intervals (CI) for associations between PA patterns and mortality. Non-linearity was accounted for by restricted cubic splines with four knots at the 0.05, 0.35, 0.65, and 0.95 quantiles. Departure from linearity was tested for all variables by testing the coefficient of the second and third spline transformation equal to zero [26]. Proportional hazard assumptions were checked using Schoenfeld residuals and visually. We conducted several sensitivity analyses to evaluate the robustness of our results. Specifically, we excluded deaths that occurred within two years after accelerometry assessment; excluded participants with prevalent CVD and/or diabetes; performed a stratified analysis among participants with prevalent CVD and/or diabetes; and used smoking intensity (pack years) and alcohol use intensity (grams per day) as covariates [27]. The influence of potential disruptions of the circadian rhythm through shift work was investigated by adjusting our model for this covariate. We examined interactions between fPCs and sedentary behavior and age. To investigate the robustness of our derived PA patterns, we tested different fPCA hyperparameters (kernel smoother and bandwidths) and correlations with accelerometer–derived proportions of sleep, sedentary time, and moderate–to–vigorous activity.

Cox regression was conducted using the *rms* package [28]. All data processing and statistical analyses were performed using R 4.2.2 [29].

## Results

We obtained PA patterns from 96,351 participants (56.3% female). Participants were 61.9 years old at accelerometry assessment. The median follow-up time was 6.9 years, during which 2,850 participants (3.0%) died (Table 1). The average daily ENMO was 28.0 ± 8.1 m*g*. There was no meaningful deviation for those who were excluded due to missing covariate data (26.9 ± 8.4 m*g*). Baseline characteristics for excluded participants did not differ from the population for analysis (Online Resource 5).

**Table 1:** Descriptive baseline characteristics

Variable	PA pattern sample (*N* = 96,351)	Mortality sample (*N* = 84,877)
Sex (%)		
*Female*	54,272 (56.3)	47,081 (55.5)
*Male*	42,079 (43.7)	37,796 (44.5)
Age at baseline (sd)	56.16 (7.82)	55.84 (7.83)
Age at accelerometry (sd)	61.86 (7.85)	61.51 (7.86)
Age at exit (sd)	68.67 (7.78)	68.35 (7.79)
Body mass index (sd)	26.72 (4.53)	26.70 (4.51)
Diet score (sd)	3.71 (1.33)	3.70 (1.33)
Socio-economic status (sd)	-1.73 (2.82)	-1.72 (2.82)
Sedentary behavior (sd)	4.29 (2.46)	4.29 (2.47)
Smoking status (%)		
*Never*	54,872 (57.0)	48,947 (57.7)
*Former*	34,574 (35.9)	30,069 (35.4)
*Current*	6,660 (6.9)	5,861 (6.9)
Pack years of smoking (sd)	20.25 (16.66)	6.49 (13.24)
Alcohol drinking status (%)		
*Never*	2,789 (2.9)	2,455 (2.9)
*Former*	2,654 (2.8)	2,318 (2.7)
*Current*	90,831 (94.3)	80,104 (94.4)
Alcohol intake in grams/day (sd)	16 (17)	16 (17)
Qualifications (%)		
*College or university degree*	41,407 (43.0)	37,088 (43.7)
*A levels/AS levels or equivalent, NVQ or HND or HNC or equivalent, Other professional qualifications*	22,599 (23.5)	20,101 (23.7)
*O levels/GCSEs or equivalent, CSEs or equivalent*	23,419 (24.3)	20,812 (24.5)
*None of the above*	7,973 (8.3)	6,876 (8.1)
Diabetes (%)		
*No*	93,737 (97.3)	82,787 (97.5)
*Yes*	2,614 (2.7)	2,090 (2.5)
Cardiovascular disease (%)		
*No*	91,634 (95.1)	80,920 (95.3)
*Yes*	4,717 (4.9)	3,957 (4.7)

The PA pattern sample consists of all participants with valid accelerometry data (those with missing body mass index were excluded). For the mortality sample, we excluded subjects with pre-accelerometry cancer and those with missing covariates

### Physical activity patterns

**Fig. 1:**
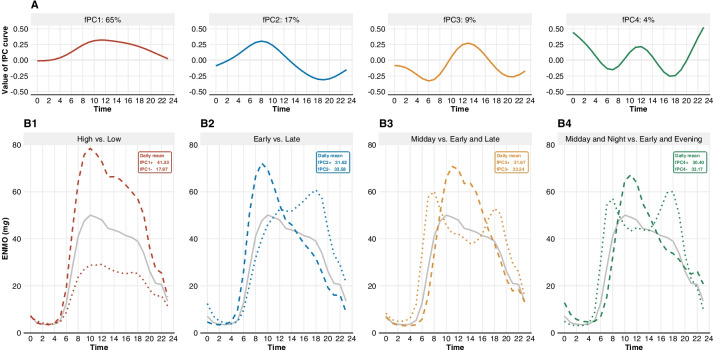
**a** The first four eigenfunctions **b** The average daily time course of PA. The solid grey line represents the population PA average (ENMOs in m*g*), the dashed line represents the positive scorers (at least one standard deviation away from the mean score), and the dotted line represents the negative scorers (at least one standard deviation away). Daily average ENMOs were similar for positive and negative scores on fPC2, fPC3, and fPC4 as well as between these fPCs

The fPCA revealed four fPCs that accounted for 95.8% of the total variance in the temporal distribution of PA during the day (Fig. 1A). No clear elbow was visible and further eigenfunctions showed no patterns that provided interpretable added value. The first fPC (fPC1) explained 65.5% of the variability denoting overall PA during the day. The second component fPC2 accounted for 17.0% of the variance depicting the contrast between early and late hours. The third component fPC3 explained 9.0% of the variance depicting the contrast of midday and early/late hours. A similar pattern was found for fPC4 (4.3% explained variance), except that fPC4 represented the contrast of midday/night and morning/evening hours.

For better interpretability, we focused on participants who scored positive (> 1 SD above the mean) or negative (< 1 SD below the mean) on a given component (Table 2). Positive scores on fPC1 were related to increased PA levels between 6AM–10PM. Positive scores on fPC2 showed early PA (8AM–12PM); on fPC3 midday PA (10AM–4PM); and on fPC4 midday PA (10AM–4PM) and nighttime PA (12AM–4AM) (Fig. 1B). Negative scores were related to inverse patterns (Table 2).

**Table 2:** Description of PA patterns

fPC	Score	Abbreviation	Description	Time period
			Participants showed…	Approximately
fPC1	positive	fPC1 +	higher overall PA	06AM – 10PM
fPC1	negative	fPC1–	lower overall PA	06AM – 10PM
fPC2	positive	fPC2 +	higher early day PA	08AM – 12PM
fPC2	negative	fPC2–	higher late day PA	06PM – 12AM
fPC3	positive	fPC3 +	higher midday PA	10AM – 04PM
fPC3	negative	fPC3–	higher early day and late day PA	08AM – 12PM and 06PM – 12AM
fPC4	positive	fPC4 +	higher midday and nighttime PA	10AM – 04PM and 12AM – 04AM
fPC4	negative	fPC4–	higher early day and evening PA	08AM – 12PM and 04PM – 08PM

Sensitivity analyses showed that results were robust for changes in the parameters used to determine fPCs. Alternatively, when an Epanechnikov kernel for smoothing was used, the explained variability was smaller for fPC1 (~ 16% decrease) but higher for fPC3 and fPC4, and more than four components were necessary to reach the 95% threshold (Online Resource 6a). Nevertheless, the shape of the first four eigenfunctions remained similar (Online Resource 6b). Variation in the bandwidths of the kernel smoothers did not affect the explained variance. Correlations between fPCs and accelerometer-derived PA variables were rather weak (Online Resource 6c).

### Mortality

**Fig. 2:**
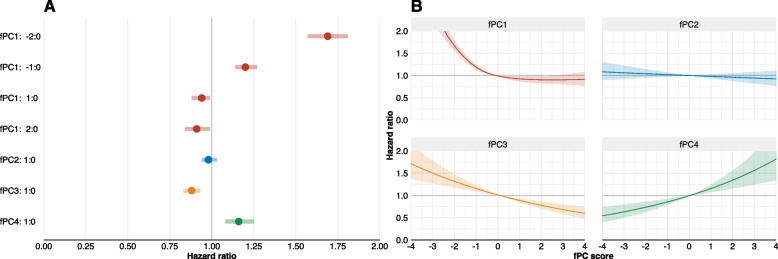
**a** HRs are reported for a one score increase except for fPC1, where HRs for a score of -2, -1, + 1, and + 2 compared to 0 are reported **b** (Non-)linear relation of continuous fPC scores and hazard ratios

We noted a strong non-linear relation of fPC1 to mortality (non-linear term *p* < 0.001). Negative scores (low overall PA) were associated with increased mortality. With a loading score of zero as the reference, fPC1 scores of -2 and -1 were related to elevated mortality, with HRs of 1.69 (95% CI: 1.57–1.81, *p* < 0.001) and 1.20 (95% CI: 1.14–1.27, *p* < 0.001), respectively. Conversely, high overall PA was associated with reduced mortality, for scoring + 2 (HR = 0.91; 95% CI: 0.84–0.99, *p* < 0.001) and + 1 (0.94; 95% CI: 0.88–0.99, *p* < 0.001). We found no association between fPC2 (early day versus late day PA) and mortality (HR for a 1-unit score increase 0.98; 95% CI: 0.94–1.03, *p* = 0.395). fPC3 (midday versus early and late day PA) was linearly associated with decreased mortality – for a 1-unit score increase, the HR was 0.88 (95% CI: 0.83–0.93, *p* < 0.001). An association in the opposite direction was apparent for fPC4, i.e., midday and nighttime PA versus early and evening PA (HR for a 1-unit score increase 1.16; 95% CI: 1.08–1.25, *p* = 0.001). Figure 2A displays the HRs for specific fPC loading scores in relation to the reference score zero. Figure 2B shows the (non-)linear relation of the component scores to all-cause mortality.

In addition, in a sensitivity model we examined the impact of smoking intensity (pack years) and alcohol intensity (grams per day) and found no deviation from our main results (Table 3). The associations also remained apparent when using another kernel smoother, with non-significant estimates for fPC4 (Online Resource 6d). Further, we excluded deaths within two years after accelerometry (*N* = 340) and the results were materially unaltered. Neither did the exclusion of participants with prevalent CVD and/or diabetes (5,618 exclusions) impact the results, nor restricting the analysis to participants with these comorbidities (*N* = 5,618) (Online Resource 7). Also, accounting for shift work did not alter the main results (*N* = 53,519) (Online Resource 8). Lastly, we examined interactions between fPC scores and sedentary behavior and age and none of the interaction terms were statistically significant (Online Resource 9).

**Table 3:** Hazard ratios of the four principal components for all-cause mortality

Component	Main model (*N* = 84,877) HR (95% CI)	Sensitivity model (*N* = 62,771) HR (95% CI)
fPC1		
-2:0	1.69 (1.57–1.81)	1.77 (1.63–1.93)
-1:0	1.20 (1.14–1.27)	1.22 (1.14–1.30)
1:0	0.94 (0.88–0.99)	0.94 (0.87–1.00)
2:0	0.91 (0.84–0.99)	0.92 (0.84–1.02)
Overall *p*-value	6.25 × 10^–56^	3.43 × 10^–50^
fPC2	0.98 (0.94–1.03)	0.98 (0.93–1.04)
Overall *p*-value	0.395	0.554
fPC3	0.88 (0.83–0.93)	0.89 (0.83–0.95)
Overall *p*-value	2.02 × 10^–6^	3.43 × 10^–7^
fPC4	1.16 (1.08–1.25)	1.15 (1.05–1.26)
Overall *p*-value	1.08 × 10^–5^	0.003

HRs are reported for a one score increase except for fPC1, where HRs for a score of -2, -1, + 1, and + 2 compared to 0 are reported. The main model was stratified by sex and study center and was further adjusted for prevalent CVD and diabetes, smoking status, alcohol consumption status, socio-economic status, education level, sedentary behavior, and diet. The sensitivity model was adjusted for pack years of smoking and alcohol grams per day instead of smoking status and alcohol consumption status

## Discussion

We derived novel circadian PA patterns using fPCA and uncovered four eigenfunctions that explained almost the entire variability of 24 h-accelerometry data in the UK Biobank. These patterns described the time course of activity throughout the day and differences between morning and evening hours. We found that three fPCs were associated with mortality. Positive fPC1 scores representing high overall PA were associated with decreased mortality, whereas negative fPC1 scores reflecting low overall PA displayed higher mortality. Negative scores on fPC3, signifying the combination of early day and late day PA were related to increased mortality, whereas positive fPC3 scores representing midday PA were associated with lower mortality. Moreover, positive fPC4 scores capturing midday and nighttime PA were associated with higher mortality, whereas negative fPC4 scores symbolizing the combination of early day and evening time PA were related to lower mortality. By comparison, fPC2 was unrelated to mortality.

Our results point towards the importance of overall PA for longevity. Inadequate levels of PA increase the risk of premature mortality, whereas high PA levels are associated with lower risk of early death [30, 31]. Our findings regarding fPC1 support this evidence. Specifically, lower fPC1 scores were associated with greater mortality hazard, and scoring highly positive on fPC1 was associated with lower premature mortality. The inverse relation of fPC1 to mortality was attenuated above a score of + 2, which mirrors the World Health Organization guidelines on PA and sedentary behavior stating that the beneficial effects of PA diminish at higher levels [32].

The timing of PA has been discussed in terms of the biological response to exercise, e.g., decreased blood glucose levels and activated metabolic pathways for PA at certain hours [33, 34]. Nevertheless, there is no consistent evidence that diurnal timing of PA matters for health [35]. This might be explained by the challenging harmonization between studies along with the neglect of nighttime PA, highlighting the importance of analyzing device-based assessed patterns. Our patterns revealed insights into the temporal distribution of activity that go beyond the general time course of PA. Positive fPC3 scores (fPC3 + ; one peak at midday) and negative fPC4 scores (fPC4–; two peaks, morning and evening) showed above-average activity levels and were related to decreased mortality. These results suggest that it is not the shape of the temporal distribution of PA that matters if a certain minimum level of (above-average) PA is achieved. Surprisingly, our results for fPC3– suggest that early and late day PA are associated with increased mortality, which somewhat contradicts the results for fPC4–. Possibly, higher PA during typical sleeping hours (e.g., 11PM-06AM) contributes to higher mortality (as also shown by fPC4 +). However, these associations may be confounded by occupation [36], lifestyle factors [37], or genetics [38]. Furthermore, subclinical disease may cause sleep disruption and slightly increased activity levels at night, suggesting potential reverse causation. However, the exclusion of deaths within the first two years after the PA assessment did not impact these results. Of note, a study published in 2022 clustered UKB accelerometer data and found that nighttime activity was associated with increased CVD incidence compared to morning PA [39]. In another study, published in 2023, the authors reported decreased mortality for midday–afternoon PA compared to morning PA, which our findings support (fPC3 +); however, in their study, evening PA (5PM–12AM) was not associated with mortality [40].

We present novel analyses of PA data using the residuals of raw accelerometry-based PA metrics. Nevertheless, the shapes of our PA patterns are similar to previous applications of fPCA to accelerometry data [9–11, 13, 14], which provides confidence in the robustness of our results. One study derived four patterns (88% variance explained) among 2,976 men and found that the first component denoted overall activity, with lower quartiles showing higher mortality hazards [14]. Another study derived four fPCs (87% variance explained) and reported associations with population characteristics and self-reported health among 7,657 individuals [9]. We greatly expand on these findings by presenting four fPCs explaining 96% of the variability of the data on 96,361 participants. Moreover, we used ENMOs and thus overcame the limitations of summary counts. We also used different kernel smoothers and bandwidths in sensitivity analyses to provide more robust results. Finally, we did not use the accelerometer data directly as input for the fPCA, but instead used the residuals, which were *a priori* adjusted for potential major confounders.

### Strengths and limitations

Our study has some limitations. First, at least two years elapsed between measurement of covariates at baseline and accelerometer measurement. During this time, covariates might have changed. However, registry data on prevalent cancer as well as hospital inpatient data on CVD and diabetes were available until the accelerometry measurement. Additionally, it is possible that participants altered their behavior because they were aware of wearing an accelerometer (‘reactivity’) [41]. Differential misclassification of the exposure could lead to biased associations with the outcome. Ultimately, the general limitations of accelerometry remain, including lack of data on context, which limits the interpretation and comparability of our results with context-based measurements. Having averaged participants’ PA over several days, we may have missed single intense PA bouts that could indeed be of particular value for health.

Notwithstanding these limitations, we were able to gain novel insights. The valuable information of the temporal distribution of PA is underutilized, with only few studies examining PA patterns [42]. We addressed this knowledge gap by conducting a robust study of PA patterns based on a large sample and associations with mortality. By using an unsupervised approach, we made no *a priori* assumptions. Our patterns were robust to different fPCA settings (smoothing kernel, varying bandwidths) and covariate modelling. Compared with previous research, we examined a significantly larger sample as well as a longer time period, and hence, presented stable effect estimates with smaller CIs. While previous accelerometer studies were based on summary statistics, we used a metric derived from raw data, which facilitates comparability and interpretability.

## Conclusion

Our study addressed a gap in previous literature regarding the temporal course of PA and its association with mortality. We found novel circadian patterns of PA using fPCA. These patterns were defined as the time course of PA over 24 h. Four eigenfunctions explained most of the variation in the data and the patterns were related to all-cause mortality. Our results indicate that it is less important during which daytime hours one is active but rather, that engaging in a minimum level of PA is associated with decreased mortality. Future studies need to confirm the validity and robustness of our methods and results. Finally, contextual information such as activity type would be of additional value.

### Supplementary Information

**Additional file 1: S1.** The Euclidean norm minus one (ENMO). **S2.** Directed Acyclic Graph. **S3.** Covariates. **S4.** Flowchart for participant inclusion. **S5.** Descriptive baseline characteristics of excluded participants. **S6.** A: Sensitivity fPCA with different bandwidth estimations and kernel smoothers. B: The first four eigenfunctions (A) and positive and negative scorers (B) when using an Epanechnikov kernel. C: Correlations between the four eigenfunctions and accelerometer-derived sleep, sedentary time, and moderate-to-vigorous physical activity. D Hazard ratios when using an Epanechnikov kernel. **S7.** Cox models without deaths within 2 years after accelerometry and without prevalent CVD and/or diabetes and restricted to participants with prevalent CVD and/or diabetes. **S8.** Cox models with shift work as additional covariate. **S9.** Interactions between fPCs and age groups and sedentary behavior.

## Data Availability

The data that support the findings of this study are available from the UK Biobank but restrictions apply to the availability of these data, which were used under license for the current study, and so are not publicly available. Researchers will need to apply to access the UK Biobank database at the following link: https://www.ukbiobank.ac.uk/enable-your-research/apply-for-access.

## Abbreviations

CI: Confidence interval
CVD: Cardiovascular disease
ENMO: Euclidean norm minus one
fPCA: Functional principal component analysis
HR: Hazard ratio
m*g*: Miligravity units
PA: Physical activity
PACE: Principal analysis by conditional estimation
SD: Standard deviation
UKB: UK Biobank
