# Supplementary material for: Association between circadian physical activity patterns and mortality in the UK Biobank
Source: Int J Behav Nutr Phys Act. 2023 Sep 1;20:102. doi: 10.1186/s12966-023-01508-z (PMC10472628; doi:10.1186/s12966-023-01508-z)
Supplement: Supplementary file 1 — Additional file 1: S1. The Euclidean norm minus one (ENMO). S2. Directed Acyclic Graph. S3. Covariates. S4. Flowchart for participant inclusion. S5. Descriptive baseline characteristics of excluded participants. S6. A: Sensitivity fPCA with different bandwidth estimations and kernel smoothers. B: The first four eigenfunctions (A) and positive and negative scorers (B) when using an Epanechnikov kernel. C: Correlations between the four eigenfunctions and accelerometer-derived sleep, sedentary time, and moderate-to-vigorous physical activity. D Hazard ratios when using an Epanechnikov kernel. S7. Cox models without deaths within 2 years after accelerometry and without prevalent CVD and/or diabetes and restricted to participants with prevalent CVD and/or diabetes. S8. Cox models with shift work as additional covariate. S9. Interactions between fPCs and age groups and sedentary behavior. [file 12966_2023_1508_MOESM1_ESM.docx]

# Association between circadian physical activity patterns and mortality in the UK Biobank

Michael J. Stein, MS; Hansjörg Baurecht, PhD; Anja M. Sedlmeier, PhD; Julian Konzok, PhD; Patricia Bohmann, MS; Emma Fontvieille, MS; Laia Peruchet-Noray, MS; Jack Bowden, PhD; Christine M. Friedenreich, PhD; Béatrice Fervers, MD, PhD; Pietro Ferrari, PhD; Marc J. Gunter, PhD; Heinz Freisling, PhD; Michael F. Leitzmann, MD, PhD; Vivian Viallon, PhD; Andrea Weber, PhD

Corresponding author: Michael J. Stein, Department of Epidemiology and Preventive Medicine, University of Regensburg, Franz-Josef-Strauss Allee 11, 93057 Regensburg, Germany, Email: michael.stein@ukr.de

# Supplemental Materials

S1 The Euclidean norm minus one (ENMO)

S2 Directed Acyclic Graph

S3 Covariates

S4 Flowchart for participant inclusion

S5 Descriptive baseline characteristics of excluded participants

S6A Sensitivity fPCA with different bandwidth estimations and kernel smoothers

S6B The first four eigenfunctions (A) and positive and negative scorers (B) when using an Epanechnikov kernel

S6C Correlations between the four eigenfunctions and accelerometer-derived sleep, sedentary time, and moderate-to-vigorous physical activity

S6D Hazard ratios when using an Epanechnikov kernel

S7 Cox models without deaths within 2 years after accelerometry and without prevalent CVD and/or diabetes and restricted to participants with prevalent CVD and/or diabetes

S8 Cox models with shift work as additional covariate

S9 Interactions between fPCs and age groups and sedentary behavior

**S1: The Euclidean norm minus one (ENMO)**

ENMOs are the Euclidean norm minus one, as described by van Hees et al. [1]. They are defined as the Euclidean norm for the three-dimensional acceleration for each time point with one gravitational unit being subtracted and negative values truncated to zero:

ENMO = $\sqrt{\text{x}^{\text{2}}\text{+}\text{y}^{\text{2}}\text{+}\text{z}^{\text{2}}}\text{-1}\text{g}$

In the UKB, ENMOs were collapsed to five-second epoch levels measured in milli gravity (m*g*) units. Hence, for each participant, up to approximately 120,000 ENMOs could be measured over the 7-day period.

1. van Hees VT, Gorzelniak L, Dean León EC, Eder M, Pias M, Taherian S, et al. Separating Movement and Gravity Components in an Acceleration Signal and Implications for the Assessment of Human Daily Physical Activity. PLOS ONE. 2013;8(4):e61691.

**
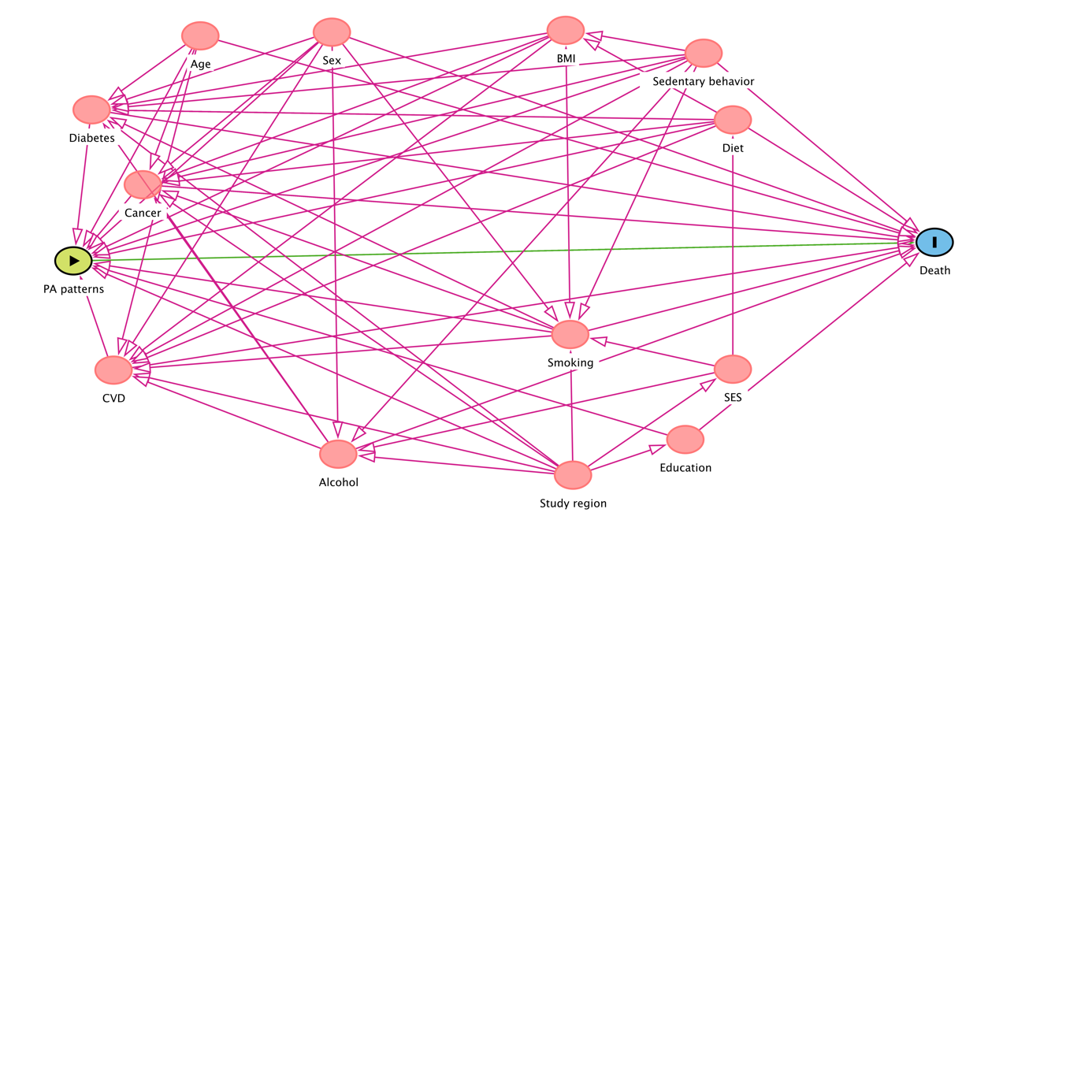
S2: Directed Acyclic Graph**

Abbreviations: PA – Physical activity, BMI – Body mass index, CVD – Cardiovascular disease, SES – Socio-economic status.

**S3: Covariates**

The main model was stratified by sex and study center and was further adjusted for prevalent type-2-diabetes and prevalent cardiovascular disease (defined as first non-fatal events of coronary artery disease, i.e., chronic ischaemic heart disease (ICD-10 I25), strokes (I60-64), and atrial fibrillation (I48)), smoking status (never; former; current), alcohol consumption status (never; former; current), socio-economic status (Townsend Index of Deprivation), education level (college or university degree; A levels/AS levels or equivalent, NVQ or HND or HNC or equivalent, other professional qualifications; O levels/GCSEs or equivalent, CSEs or equivalent; none of the above), sedentary behavior (sum of time spent watching TV, using a computer during leisure time, and driving for transportation), and diet (healthy diet score [1] adjusted to a 0–7 scale). Prevalent diabetes and cardiovascular disease were obtained from hospital inpatient data until the date of accelerometry. The other covariates were measured at baseline (2006–2010), i.e., before accelerometry assessment.

Sedentary behavior was weakly correlated with accelerometry-derived sedentary time (r=0.17). This might be explained by the fact that a cutoff-based measure derived from accelerometry does not well-reflect sedentary behavior as defined by time spent watching TV, using the computer, or driving.

1. Lourida I, Hannon E, Littlejohns TJ, Langa KM, Hyppönen E, Kuźma E, et al. Association of Lifestyle and Genetic Risk With Incidence of Dementia. JAMA. 2019;322(5):430-7.

**
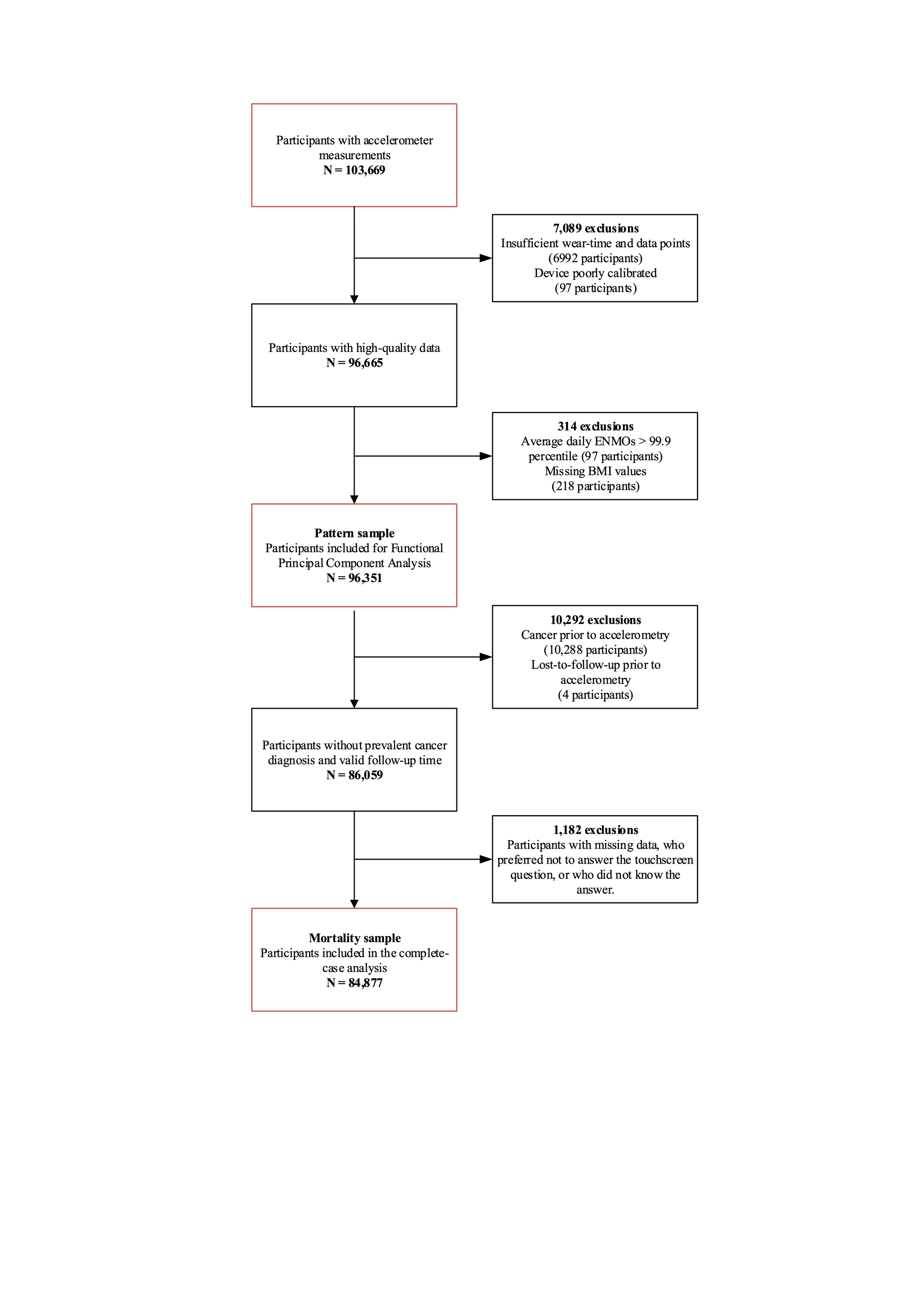
S4: Flowchart for participant inclusion**

**S5: Descriptive baseline characteristics of excluded participants**

| **Variable** | **Excluded due to poor accelerometry data**  **(N = 6,960)** | **Excluded due to missing covariate data**  **(N = 1,326)** |
| --- | --- | --- |
| Sex (%) |  |  |
| *Female* | 3,821 (54.90) | 743 (56.03) |
| *Male* | 3,139 (45.10) | 583 (43.97) |
| Age at baseline (sd) | 54.69 (7.99) | 56.46 (7.97) |
| Age at accelerometry (sd) | 60.36 (7.99) | 63.37 (7.88) |
| Age at exit (sd) | 67.14 (7.92) | 70.01 (7.80) |
| Body mass index (sd) | 27.05 (4.77) | 26.92 (4.68) |
| Diet score (sd) | 3.70 (1.32) | 3.64 (1.32) |
| Socio-economic status (sd) | -1.47 (2.96) | -1.44 (3.00) |
| Sedentary behavior (sd) | 4.38 (2.60) | 4.08 (2.55) |
| Smoking status (%) |  |  |
| *Never* | 3,871 (55.62) | 599 (45.17) |
| *Former* | 2,481 (35.65) | 383 (28.88) |
| *Current* | 591 (8.49) | 99 (7.47) |
| Pack years of smoking (sd) | 19.79 (16.85) | 24.48 (17.66) |
| Alcohol drinking status (%) |  |  |
| *Never* | 194 (2.79) | 49 (3.70) |
| *Former* | 191 (2.74) | 39 (2.94) |
| *Current* | 6,570 (94.4) | 1,161 (87.56) |
| Alcohol intake in grams/day (sd) | 17 (17) | 17 (18) |
| Qualifications (%) |  |  |
| *College or university degree* | 3,066 (44.05) | 130 (9.80) |
| *A levels/AS levels or equivalent, NVQ or HND or HNC or equivalent, Other professional qualifications* | 1,636 (23.51) | 80 (6.03) |
| *O levels/GCSEs or equivalent, CSEs or equivalent* | 1,726 (24.8) | 82 (6.18) |
| *None of the above* | 461 (6.62) | 81 (6.18) |
| Diabetes (%) |  |  |
| *No* | 6,720 (96.55) | 1,273 (96.00) |
| *Yes* | 229 (3.29) | 53 (4.00) |
| Cardiovascular disease (%) |  |  |
| *No* | 6,648 (92.52) | 1,248 (94.12) |
| *Yes* | 312 (4.48) | 78 (5.88) |
| The proportions of participants excluded due to missing covariate data do not necessarily sum up to 100% due to missing data. | | |

**S6A: Sensitivity fPCA with different bandwidth estimations and kernel smoothers**

|  | **Fraction of variance explained** | | | | | | | | | |  |
| --- | --- | --- | --- | --- | --- | --- | --- | --- | --- | --- | --- |
|  | *K* | | | | | | | | | |  |
|  | 1 | 2 | 3 | 4 | | 5 | 6 | 7 | 8 | 9 | |
| **Gaussian** | | | | |  | | | | | | |
| Default | 65.49% | 17.01% | 9.00% | 4.32%***** | | 2.95% | 0.73% | - | - | - | |
| GCV | 70.29% | 13.80% | 9.19% | 3.25%* | | 2.21% | 0.86% | - | - | - | |
| GMeanGCV | 66.29% | 15.57% | 9.37% | 4.16%* | | 3.16% | 0.94% | - | - | - | |
| **Epanechnikov** | | | | |  | | | | | | |
| Default | 50.33% | 16.62% | 13.82% | 6.74% | | 4.78% | 2.97%* | 2.26% | 1.08% | 0.97% | |
| GCV | 49.47% | 15.28% | 13.26% | 9.22% | | 5.21% | 2.84%* | 2.15% | 1.16% | 0.94% | |
| GMeanGCV | 49.80% | 16.30% | 13.58% | 7.22% | | 5.29% | 2.98%* | 2.25% | 1.10% | 1.02% | |
| *Cumulative fraction of variance explained above 95%.  Note: Default refers to the default settings of the fPCA function in fdapace. GCV is Generalized Cross-Validation; GMeanGCV is Geometric Mean and GCV. | | | | | | | | | | | |

**
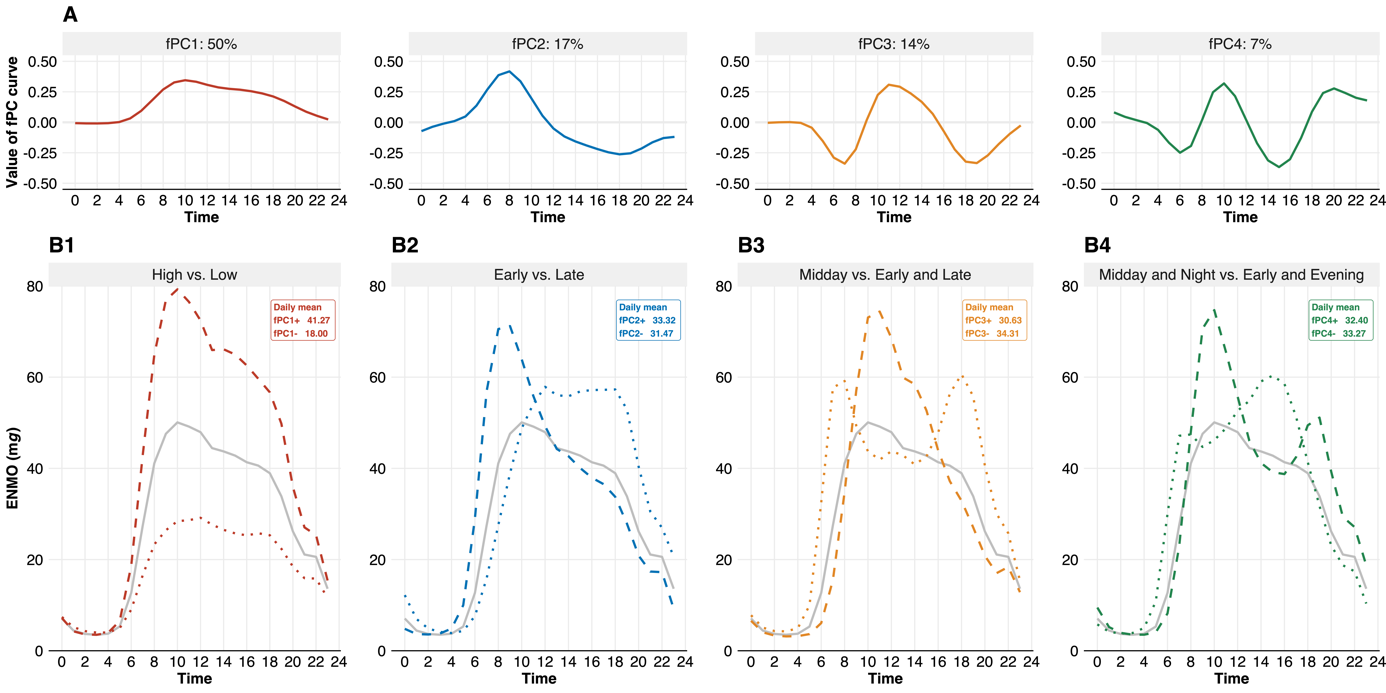
S6B: The first four eigenfunctions (A) and positive and negative scorers (B) when using an Epanechnikov kernel**

**S6C: Correlations between the four eigenfunctions and accelerometer-derived sleep, sedentary time, and moderate-to-vigorous physical activity**

| fPC | Overall sleep  percentage | Overall sedentary time percentage | Overall moderate-to-vigorous percentage |
| --- | --- | --- | --- |
| fPC1 | -0.23 | -0.55 | 0.55 |
| fPC2 | 0.10 | 0.01 | 0.04 |
| fPC3 | 0.29 | -0.19 | 0.06 |
| fPC4 | 0.06 | 0.00 | -0.07 |
| Note: Accelerometer-derived variables are the overall average proportion of time spent doing the respective activity. | | | |

**
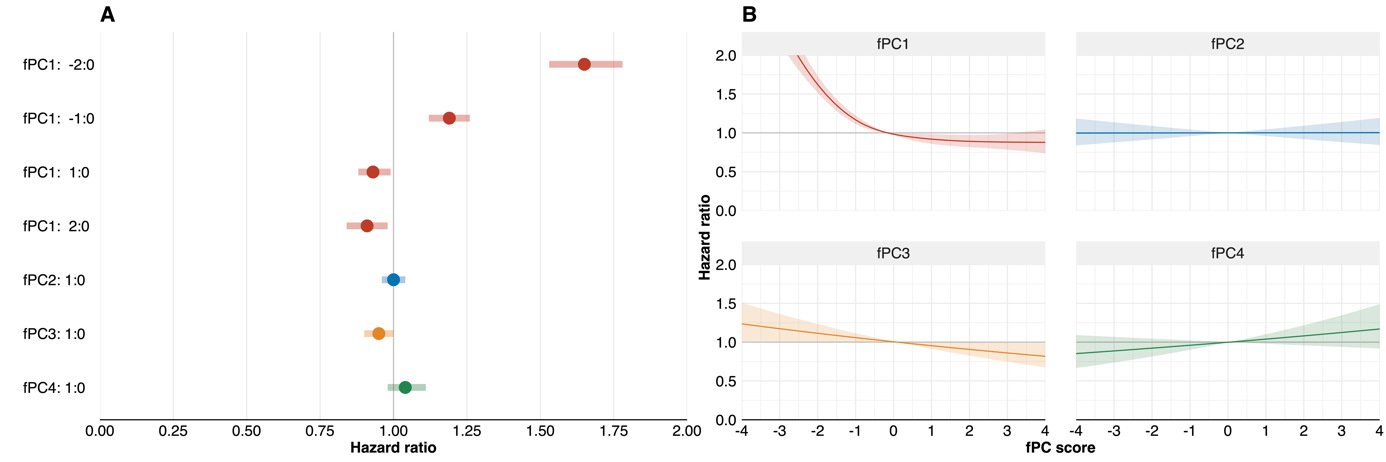
S6D: Hazard ratios when using an Epanechnikov kernel**

**S7: Cox models without deaths within 2 years after accelerometry and without prevalent CVD and/or diabetes and restricted to participants with prevalent CVD and/or diabetes**

| **Component** | **Model with deaths within 2 years excluded**  **HR (95% CI)**  N=84,537;  Cases=1,726 | **Model with prevalent CVD and/or diabetes excluded**  **HR (95% CI)**  N=79,259; Cases=1,695 | **Model with only prevalent CVD and/or diabetes**  **HR (95% CI)**  N=5,618;  Cases=371 |  |
| --- | --- | --- | --- | --- |
| fPC1 |  |  |  |  |
| *-2:0* | 1.59 (1.46–1.72) | 1.68 (1.55­–1.82) | 1.73 (1.47­–2.04) |  |
| *-1:0* | 1.18 (1.11–1.25) | 1.20 (1.13–1.28) | 1.21 (1.07–1.37) |  |
| *1:0* | 0.94 (0.88–1.00) | 0.93 (0.87–1.00) | 0.93 (0.81–1.07) |  |
| *2:0* | 0.91 (0.83–1.00) | 0.91 (0.83–1.00) | 0.90 (0.74–1.09) |  |
| *Overall p-value* | 6.78 × 10^-36^ | 8.06 × 10^-43^ | 7.45 × 10^-17^ |  |
| fPC2 | 0.96 (0.92–1.01) | 0.98 (0.93–1.03) | 0.95 (0.86–1.05) |  |
| *Overall p-value* | 0.150 | 0.500 | 0.3319 |  |
| fPC3 | 0.90 (0.85–0.96) | 0.90 (0.85–0.96) | 0.78 (0.86–0.89) |  |
| *Overall p-value* | 0.001 | 0.001 | 0.003 |  |
| fPC4 | 1.10 (1.01–1.20) | 1.13 (1.04–1.23) | 1.19 (1.00–1.41) |  |
| *Overall p-value* | 0.032 | 0.003 | 0.0440 |  |

**S8: Cox models with shift work as additional covariate**

| **Component** | **Hazard ratio (95% CI)**  N=53,519; Cases=842 |
| --- | --- |
| fPC1 |  |
| *-2:0* | 1.74 (1.55–1.95) |
| *-1:0* | 1.21 (1.11–1.31) |
| *1:0* | 0.95 (0.86–1.04) |
| *2:0* | 0.94 (0.83–1.07) |
| *Overall p-value* | 3.26 × 10^-25^ |
| fPC2 | 1.02 (0.95–1.09) |
| *Overall p-value* | 0.6208 |
| fPC3 | 0.92 (0.85–0.99) |
| *Overall p-value* | 0.0287 |
| fPC4 | 1.14 (1.02–1.27) |
| *Overall p-value* | 0.0197 |

**S9: Interactions between fPCs and age groups and sedentary behavior**

| **Component** | **P value for age group interaction** | **P value for sedentary behavior interaction** |
| --- | --- | --- |
| fPC1 | 0.1210 | 0.5076 |
| fPC2 | 0.4095 | 0.3497 |
| fPC3 | 0.1007 | 0.7402 |
| fPC4 | 0.7442 | 0.2671 |
